# Seizures and Basal Ganglia Calcification Caused by Hypoparathyroidism

**DOI:** 10.7759/cureus.7888

**Published:** 2020-04-29

**Authors:** Tabinda Nageen, Syed Zaki Muhammad, Ammarah Jamal

**Affiliations:** 1 Pediatrics, Civil Hospital Karachi, Dow University of Health Sciences, Karachi, PAK; 2 Internal Medicine, Dow Medical College, Dow University of Health Sciences, Karachi, PAK

**Keywords:** idiopathic hypoparathyroidism, seizures, basal ganglia calcification, hypoparathyroidism

## Abstract

Seizures, not a typical feature of hypoparathyroidism, may present along with myalgia and tetany in patients of hypoparathyroidism. Thorough history and examination, derangement of biochemical parameters such as hypocalcemia, hyperphosphatemia, and inappropriately low levels of parathyroid hormone, along with basal ganglia calcification as seen on imaging, lead to the diagnosis of hypoparathyroidism in a 10-year-old child who presented to us. Treatment with calcium and active vitamin D significantly improved his condition. In this report, we discuss the presentation and treatment of hypoparathyroidism. Early detection and follow-up at clinically appropriate intervals is important to avoid complications.

## Introduction

Basal ganglia calcification (BGC) is a rare phenomenon in children [1]. It is, however, present in >70% of patients with idiopathic hypoparathyroidism [2,3]. BGC itself is an unlikely cause of seizures; however, it relates to seizures in hypoparathyroidism [3]. Hypoparathyroidism is a potentially treatable cause of this calcification. If hypoparathyroidism is left undiagnosed or untreated, it can lead to multiple consequences, including renal disease, intracranial calcifications, seizures, neuropsychiatric disease, and cataracts [4]. Hence, it should be ruled out by investigating serum levels of calcium, phosphate, and parathyroid hormone (PTH) [5]. Hypoparathyroidism is characterized by hypocalcemia, hyperphosphatemia, and low or inappropriately normal levels of PTH [6]. Here we report a case of a 10-year-old boy who was being treated for epilepsy, but was ultimately diagnosed as a case of idiopathic hypoparathyroidism.

## Case presentation

We report the case of a 10-year-old boy who presented to us at the outpatient department with twitching of perioral muscles and carpopedal spasms for the past day. The patient had long-standing history of tingling in fingers, muscle spasms, and pain mostly involving distal muscles of the limbs, on and off for the past five years, approximately once a month, with no aggravating or relieving factor known to the patient. Each episode lasted for few hours.

Earlier he was hospitalized at the age of five years with complaints of afebrile, generalized tonic-clonic seizures. The neurophysician suspected epilepsy and prescribed antiepileptic drugs (AEDs) (valproic acid). The patient continued to experience one to two episodes of seizures per month, for which initially the physician had gradually increased the valproic acid dosage, and later another AED, levetiracetam, was prescribed along valproic acid. He was at presentation taking both the drugs.

There is no history of breathing difficulties, headaches, mood swings, palpitations, vertigo, vomiting, diarrhea, constipation, urinary complaints, previous surgery, or blood transfusion. He was consuming 70% of required calories without adequate daily intake of milk or dairy products. The patient was the third child of non-consanguineous marriage, with no significant perinatal complications, and was immunized completely according to Expanded Program of Immunization (EPI). Family history was insignificant for epilepsy or other chronic illnesses. The patient was a school going child with an appropriate developmental history.

On examination at the time of admission, the patient was found to have a height of 136 cm (25th percentile), a weight below 5th percentile, with no dysmorphic features, sitting comfortably, and having normal vital signs including blood pressure. Neurological examination revealed an alert child oriented to time, place, and person, with normal sensory and motor functions and normal fundoscopy. Chvostek’s and Trousseau’s signs were found to be positive. Otherwise, cardiac, respiratory, and abdominal examination did not reveal any significant findings.

Based on the clinical picture, we suspected hypocalcemia as the probable cause of his symptoms.

Initial panel of investigations showed normal complete blood count, normal serum electrolytes, hypocalcemia (corrected calcium values of 4.1 mg/dL), and hyperphosphatemia (7.2 mg/dL), while serum vitamin D, serum magnesium, and serum creatinine levels were found to be within normal range thus excluding the renal and nutritional causes for this hypocalcemia. Further investigations revealed inappropriately normal serum PTH level (11.04 pg/mL). Urinary calcium to creatinine ratio was below normal for age (0.03 mg/mg) and 24-hour urinary calcium excretion (68 mg/24 hours) was low (Table 1).

**Table 1:** Laboratory investigations of the patient.

Laboratory Investigations	Patient’s Value	Reference Values
Sodium	134 mEq/L	135-145 mEq/L
Potassium	3.4 mEq/L	3.4-4.7 mEq/L
Chloride	92 mEq/L	90-110 mEq/L
Calcium	3.3 mg/dL	8-10 mg/dL
Phosphate	7.2 mg/dL	3-4.5 mg/dL
Vitamin D	70 ng/mL	20-30 ng/mL
Magnesium	1.8 mg/dL	1.8-3 mg/dL
Creatinine	0.7 mg/dL	0.5-1.0 mg/dL
Parathyroid hormone	11.04 pg/mL	10-60 pg/mL
Urinary calcium/creatinine ratio	0.03 mg/mg	0.10 mg/mg
24-hour urinary calcium	68 mg/24 hours	100-300 mg/24 hours

MRI brain showed bilateral BCG. Although CT of the brain is the best modality for imaging calcification, we decided to order MRI to rule out any epileptogenic lesion (ischemia, vascular malformation, structural lesion, etc.). Electroencephalography (EEG) showed slow waves and spikes bilaterally at temporal regions, predominantly on the left side.

The patient was diagnosed as a case of idiopathic hypoparathyroidism after excluding all the secondary causes. AEDs were discontinued, and intravenous calcium replacement (calcium gluconate under cardiac monitoring) was given subsequent to which the child improved. He was discharged on oral calcium along with activated form of vitamin D. Patient’s parents were counseled regarding this chronic condition and the need to maintain treatment and dietary changes throughout life.

The patient was followed up, and serum levels of calcium, phosphate, and vitamin D were monitored along with renal ultrasound to check for nephrolithiasis and nephrocalcinosis. At two years post diagnosis, the patient has been compliant and has reported no complications.

## Discussion

PTH is an important calcium-regulating hormone that is required for calcium homeostasis, absorption of calcium from the gut (via vitamin D hydroxylation), renal reabsorption of calcium, and renal clearance of phosphate [6]. Hypoparathyroidism primarily develops due to ineffective production or secretion of PTH and defects in the PTH receptor [7]. The causes of hypoparathyroidism include congenital disorders, receptor insensitivity, surgery, autoimmune disorders, radiation, infiltrative disorders, or it can be idiopathic [5,6]. Idiopathic hypoparathyroidism is diagnosed after ruling out all secondary causes [7].

Hypocalcemia, hyperphosphatemia, and low or inappropriately normal PTH levels characterize hypoparathyroidism, all the features being fulfilled by our patient. Patients with hypoparathyroidism present variably and may complain of fatigue, muscle cramps, twitching, bronchospasm, confusion, seizures, and congestive heart failure [5-7]. Our patient’s complaint was consistent with muscle spasms, twitching, and seizures. Seizures in hypoparathyroidism have been associated to hypocalcemia, which causes an increase in neuronal excitability due to reduced extracellular calcium concentration [8]. EEG of these patients may show bilateral sharp and slow wave discharges of unusually long duration due to hypocalcemia [7]. Our patient’s EEG had a similar pattern with slow waves and spikes. Electrocardiograph commonly reveals QTc interval prolongation. Hypocalcemia-associated neuromuscular irritability presents as paresthesia and tetany, and can be detected with positive Chvostek’s and Trousseau’s signs on examination as were seen in our patient [9].

Intracranial calcifications can be seen with hypoparathyroidism, the exact mechanism of which is not known; however, it is attributed to chronic disturbances in serum concentrations of calcium and phosphate [3]. The most common sites of calcification are the basal ganglia, as was the case in our patient, but calcification may also affect frontal cortex, thalamus, and cerebellum [7]. BCG in pediatric age group is pathological and may be seen in various pathologies, including endocrine disorders, congenital disorders, metabolic disorders, infections, and toxic conditions; however, hypoparathyroidism is the most common treatable cause [5].

Specific symptoms, thorough history, and detailed clinical examination lead to the diagnosis of idiopathic hypoparathyroidism. Autoimmune polyglandular syndrome 1 should be ruled out by checking for mucocutaneous candidiasis and vitiligo, none of which was present in our patient. In addition, growth retardation, congenital anomalies, hearing loss, and mental retardation suggest the possibility of genetic syndromes as causes for the disease; however, these were absent in our patient. Biochemical abnormalities in hypoparathyroidism could be caused by hypomagnesaemia, vitamin D resistance or deficiency, and renal failure, and hence these should be ruled to establish idiopathic hypoparathyroidism. These conditions were investigated and excluded. Secondary causes were ruled out to reach the diagnosis of idiopathic hypoparathyroidism [10]. 

Calcium and active vitamin D supplementation are recommended therapy for hypoparathyroidism and similar were prescribed to our patient [5-8]. Patients with hypoparathyroidism should be followed up clinically, and laboratory investigations should be ordered as clinically indicated to monitor the course of disease and register any abnormalities [7].

Through this case report, we aim to emphasize certain points. Patients with a history of seizures might be labeled as epileptic without proper investigation of causative factors and treatable causes may be overlooked, such as in this report where the child was being treated with antiepileptics. It is therefore important to rule out common causes such as electrolyte abnormalities and central nervous system infections. If further investigation is required, hypoparathyroidism should be included in the differentials of young patients with a history of seizures. Furthermore, we highlight BCG in hypoparathyroidism; such radiologic finding should warrant a differential of hypoparathyroidism. In addition, the diagnosis of hypoparathyroidism may also be missed totally with non-specific muscular pains and spasms either ignored or treated symptomatically with analgesics. 

## Conclusions

This case highlights the need to exclude all the possible organic causes of seizures before diagnosing as epilepsy as per standard definition of epilepsy. It also highlights the importance of investigating for hypoparathyroidism when BCGs are seen on imaging. Since early diagnosis and timely, effective treatment would avoid long-term complications. Clinical follow-up along with laboratory investigations at relevant intervals is recommended to monitor the disease.
